# Association of polysocial risk score, cardiovascular health status, and the risk of premature mortality: Findings from the UK Biobank

**DOI:** 10.1016/j.jnha.2025.100527

**Published:** 2025-03-08

**Authors:** Wenqi Shen, Lingli Cai, Bin Wang, Jiang Li, Ying Sun, Ningjian Wang, Yingli Lu

**Affiliations:** Institute and Department of Endocrinology and Metabolism, Shanghai Ninth People’s Hospital, Shanghai Jiao Tong University School of Medicine, Shanghai, China

**Keywords:** Polysocial risk score, Premature mortality, Cardiovascular health, Social determinants of health

## Abstract

**Background:**

Evidence of the cumulative effects of social risk factors on premature mortality is quite limited. We aimed to examine the association between cumulative social risk factors and premature mortality by constructing a polysocial risk score, and to explore the influence of cardiovascular health on this association.

**Methods:**

A polysocial risk score was constructed by summing 11 social determinants of health. A cardiovascular health (CVH) score was calculated following the algorithm of "Life’s Essential 8”. Premature mortality was defined as death at an age younger than 75. Cox proportional hazards model was conducted.

**Results:**

A total of 314,039 participants in the UK Biobank were included (median age 56.0 years, 53.1% women). During a median of 12.7 years of follow-up, 13,888 premature deaths were reported. Compared with participants who had a low polysocial risk score (≤3), participants with a high polysocial risk score (≥7) were more than twice as likely to die prematurely in the follow-up period (HR 2.18, 95% CI 2.06–2.30). Compared with participants with ideal CVH and low polysocial risk score, those with poor CVH and high polysocial risk score had the highest risk of premature mortality (HR 5.25, 95% CI 4.48–6.14). A significant interaction was found between CVH status and polysocial risk score on premature mortality risk (*P* for interaction <0.001).

**Conclusions:**

Polysocial risk score was associated with an increased risk of premature mortality, the association was exacerbated by poor CVH. Our findings indicate that limiting social inequities and encouraging people to achieve an ideal CVH are essential to reducing the burden of premature mortality.

## 1 Introduction

Despite the constantly declining mortality rate in recent decades, disproportionately higher rates of mortality were observed among socially disadvantaged populations [1,2]. Individuals facing social inequities, such as lower income or education levels, have a higher likelihood of premature death [[3], [4], [5]]. In addition to traditional measures of socioeconomic status, the influence of emerging factors has drawn the public’s attention. For instance, living alone, lacking social support, and experiencing social isolation have been associated with increased mortality risk [[6], [7], [8]]. On the other hand, proximity to green spaces and natural environments has shown protective effects on premature mortality [9]. Researchers also showed that decreasing ambient air pollution may significantly reduce the number of premature deaths in European cities [10].

The Healthy People 2030 initiative summarized five domains of key upstream drivers of health, known as social determinants of health (SDOH), including economic stability, education access and quality, health care access and quality, neighborhood and built environment, social and community context [11]. Notably, previous studies investigating the associations of SDOH with mortality and other health-related outcomes mainly involved one or a few metrics, with a focus on income, occupation, and education, while overlooking the complex interconnections of multiple risk factors.

Combinations of social risk factors are known to interact synergistically. In recent years, with extensive information on SDOH being increasingly collected through survey databases, Helen L Ford et al. proposed the idea of a polysocial risk score to evaluate the burden of adverse health outcomes at an individual level [12]. The polysocial risk score might provide a more holistic picture of one’s social status, and offer better insights into the association with health. To date, limited studies have investigated the relationship between polysocial risk score and premature mortality, and the combined relationship of polysocial risk score with lifestyles on premature mortality remained unclear. Approximately 25% of premature deaths are attributed to cardiovascular diseases (CVD). To our knowledge, no previous study has applied the newly defined metric “Life’s essential 8” by the American Heart Association (AHA) to quantify an individual’s overall cardiovascular health (CVH) and assess its impact on the social disadvantage in health.

To fill these research gaps, we aimed to examine the association of combined social risk factors, based on a polysocial risk score, with premature mortality using the UK Biobank cohort. Additionally, we investigated the extent to which cardiovascular health status, defined by “Life’s essential 8”, might affect the association between polysocial risk score and premature mortality.

## 2 Methods

### 2.1 Study population

The data of this population-based prospective study was generated from UK Biobank, detailed information on UK Biobank was published in previous reports and can be found at www.ukbiobank.ac.uk [13]. In brief, from 2006 to 2010, over half a million people aged 37–73 years old were recruited across the United Kingdom (England, Wales, and Scotland). The North West Multicenter Research Ethics Committee approved the UK Biobank study. At the time of enrollment, each participant provided written informed consent. Among the 502,415 participants in the UK Biobank, we excluded those with missing information to calculate the polysocial risk score (n = 188,376), and 314,039 participants were included in the primary analysis. In the joint association of polysocial risk score and CVH status analysis, we further excluded participants with missing information to calculate CVH score (n = 97,879), thus, a total of 216,160 participants were included in the secondary analysis (Figure S1).

### 2.2 Polysocial risk score

Based on previous literature reviews and data availability in the UK biobank, we pre-selected 15 commonly acknowledged social determinants of health that are associated with health outcomes and mortality [[14], [15], [16]]. These 15 social determinants were classified into three domains to capture the overall individual-level social risks, namely socioeconomic status, social and community context, neighborhood and living environment. A detailed list and description can be found in Table S1. Briefly, for socioeconomic status, the participants were considered at risk if they (1) had an education level below college (low educational attainment); (2) scored below the median for education quality (poor education quality); (3) had a pre-tax household income below £31,000 per year (low household income); (4) not being employed or self-employed (unemployed); (5) had a Townsend deprivation index score above the median (indicating area-level material deprivation). Regarding social and community context, the participants were considered at risk if they met the following conditions: (1) living alone; (2) lacking someone nearby whom they could confide in at least once a week (lack of social support); (3) attending group activities less frequently than once a week (social inactivity); (4) having infrequent visits with friends or family or rarely being visited by them (social isolation); (5) experiencing illness, injury, bereavement, or stress within the last two years (emotional distress); (6) having visited a psychiatrist for issues such as nerves, anxiety, tension, or depression (psychiatrist visit). For neighborhood and living environment, the participant was considered at risk if they met the following criteria: (1) residing in a neighborhood with a crime score above the median (high local crime rate); (2) having a percentage of natural land in their home location buffer below the median (indicating a lack of natural environment proximity); (3) having particulate matter air pollution (pm2.5) above the median; (4) experiencing noise pollution with a 24-h sound level above the median.

After performing a mutually adjusted regression model, 11 social determinants remained statistically associated with the risk of premature mortality (Table S2). Thus, we constructed a polysocial risk score (ranging from 0 to 11) based on these 11 factors, participants scored 1 point for each social determinant if they were considered at risk. A higher polysocial risk score indicates a greater social vulnerability. Based on the score distribution among total participants, we defined the cutoff points of 3 and 7, participants were assigned into three groups, low (≤3), intermediate (4−6) and high (≥7) social risk groups.

### 2.3 Cardiovascular health status

We used a recently updated algorithm from the AHA to assess an individual’s overall cardiovascular health [17]. The namely Life’s Essential 8 score included eight components, diet, physical activity, smoking, sleep, ethnicity, body mass index (BMI), cholesterol, blood glucose, and blood pressure. Fasting plasma glucose was employed by the AHA for assessing hyperglycemia; however, hemoglobin (HbA1c) was used to assess blood glucose in the current study to maximize the sample size [18]. The calculation of the CVH score was described in detail elsewhere [19]. Briefly, the overall CVH score ranges from 0 to 100, and it was calculated by summing the unweighted average score of the 8 component metrics. Following the recommendation of AHA, in the subgroup analysis, participants were further divided into three groups: ideal CVH: 80–100, moderate CVH: 50–79, poor CVH: 0–49.

### 2.4 Assessments of covariates

Sex, age, BMI, smoking status, alcohol consumption, diet, sleep duration, physical activity, HbA1c, non-high-density lipoprotein (non-HDL), and systolic blood pressure (SBP) were selected as potential covariates. Age was calculated based on birth date and date of participation. Ethnicity was identified using the baseline touchscreen questionnaires, since the majority of participants in the UK biobank study were white British, ethnicity was categorized as White/others. BMI was calculated by weight (kg) divided by the square of height (m^2^). Smoking status was identified from the self-reported touchscreen questionnaires as never, previous, or current. Moderate alcohol consumption was defined as up to 14 g per day for women and up to 28 g per day for men [20]. A healthy diet was defined as an adequate intake of at least 4 of the following 7 dietary components including increased consumption of fruits, vegetables, whole grains, (shell) fish, and reduced or no consumption of refined grains, processed and unprocessed meats following recommendations on dietary [21]. Adequate sleep duration was defined as sleep 7–9 h/day [22]. Regular physical activity was defined as getting moderate activity over 150 min or vigorous activity over 75 min per week or an equivalent combination [23]. The HbA1c level was measured by high-performance liquid chromatography using the VARIANT II Turbo analyzer (Bio-Rad Laboratories, UK). The n-HDL was measured by a Beckman Coulter AU5800 clinical chemistry analyzer. Systolic blood pressure was assessed via an automated sphygmomanometer (Omron, USA). History of cancer was identified by linkage to medical records based on the International Classification of Diseases-10th Revision ICD-10: C00 to D48. History of cardiovascular disease was identified based on ICD-10: I20 to 25, I48, I50, I60 to 64. The baseline history of diabetes was defined based on ICD-10 code E11 and self-reported diagnoses by doctors.

### 2.5 Definition of premature mortality

Premature mortality refers to death that occurs before the average age of death in a certain population. In the present study, premature mortality was defined as death at an age younger than 75, in accordance with previous literature and the National Health Service (NHS) of the United Kingdom [3,24]. Information on the cause of death and death date was ascertained using linkage to death certificate records in the NHS Information Center (for participants in England and Wales) and the NHS Central Register Scotland for participants in Scotland. Follow-up time was calculated from the date of participation to the date of death or Dec 31, 2021, whichever came first. The ICD-10 codes were used to identify death types, including cancer (C00-C97), cardiovascular disease (I00-I99), respiratory disease (J00-J99), digestive disease (K20-K93), and neurodegenerative diseases (F01-F03, G20-21, G30-31).

### 2.6 Statistical analysis

The baseline demographic and lifestyle characteristics of the study population were described according to the polysocial score category (low, intermediate, and high). For continuous variables, data were presented as mean ± standard deviation (SD), for categorical variables, data were presented as numbers and percentages (%). Time-to-event was calculated from participation date to the date of premature mortality or the censoring date (Dec. 31, 2021), whichever came first. Cox proportional hazard regression models were fitted to examine the association of polysocial risk score with premature mortality. The hazard ratio (HR) and 95% confidence interval (CI) were calculated with the time-to-event as the time scale. We examined the proportional hazards assumption using Schoenfeld residuals and found no significant violation. Three models were performed, model 1 was only adjusted for age (continuous) and sex (category). Model 2 was adjusted for age, sex, ethnicity (category), BMI (continuous), diet (category), smoking (category), physical activity (category), moderate drinking (category), and sleep (category). Model 3 was adjusted for all covariables in model 2 plus HbA1c (continuous), n-HDL (continuous), and SBP (continuous). For missing categorical covariates, a mode imputation approach was used. For missing continuous covariates, we imputed the mean values. We examined the dose-response relationship between the polysocial risk score and incidence of premature mortality by restricted cubic splines with knots at the 10th, 50th, and 90th percentiles of polysocial risk score. Analyses were performed with stratification by baseline history of cancer, CVD, and diabetes to test whether the effect of polysocial risk score on premature death was varied by disease status. As a secondary analysis, we restricted the analyses to participants with full data on CVH score only. We examined the association of the combination of polysocial risk score and CVH score (9 categories with low polysocial risk score and ideal CVH score as reference) with premature mortality. Statistical interaction between polysocial risk score and CVH score was tested by fitting an interaction term in the models. Multiple sensitivity analyses were carried out to assess the robustness of our findings. First, we constructed a weighted polysocial risk score considering effect size (β coefficient) for individual social risk factors derived from the adjusted model: weighted polysocial risk score = (β1 × factor1 + β2 × factor2 +…+ β11 × factor11) × (11/sum of the β coefficients) [25]. We tested the association of weighted polysocial risk score and incidence of premature mortality. Second, we excluded cases occurred within the first three years of the follow-up. Third, we further adjusted for CRP and family history of diseases in the regression model. Fourth, we further adjusted for co-morbidities in the regression model. Fifth, we conducted analyses to examine the associations between individual components of the polysocial risk score and premature mortality. All analyses were performed with SPSS, Version 25 (IBM Corporation, Armonk, NY, USA) and R, version 4.1.2 (The R Foundation for Statistical Computing, Vienna, Austria). A two-sided *P* < 0.05 was considered statistical significance.

## 3 Results

### 3.1 Baseline characteristics

The baseline characteristics of the study participants by polysocial risk score are presented in Table 1. Among a total of 314,039 participants (median age 56.0 years, 53.1% women), 41.9%, 47.1%, and 10.9% were categorized into the low, intermediate, and high social risk groups, respectively. Participants with a higher polysocial risk score were older, more likely to be women and current smokers, had higher BMI, and were less likely to exercise regularly. They also tended to have worse diet and sleep (all *P* values<0.001). A higher prevalence of diabetes, CVD, and cancer were also found among participants with higher polysocial risk scores.

**Table 1:** Baseline characteristics of participants by polysocial risk score.

		Polysocial risk score	
	Low (≤3)	Intermediate (4−6)	High (≥7)
Number of participants	131681 (41.9)	147985 (47.1)	34373 (10.9)
Age, years	54.49 ± 7.76	55.91 ± 8.19	56.58 ± 8.15
Men, n (%)	65269 (49.6)	66839 (45.2)	15267 (44.4)
Ethnicity, White, n (%)	127635 (96.9)	139582 (94.3)	31372 (91.3)
Body mass index, kg/m^2^	26.62 ± 4.21	27.47 ± 4.83	28.51 ± 5.71
Systolic blood pressure, mmHg	137.67 ± 18.83	139.06 ± 19.21	139.15 ± 19.47
HbA1c, %	5.36 ± 0.48	5.43 ± 0.57	5.56 ± 0.75
n-HDL, mg/dl	164.03 ± 37.25	164.17 ± 38.44	163.32 ± 40.33
Smoking, n (%)	8131 (6.2)	14928 (10.1)	6848 (19.9)
Moderate Alcohol consumption, n (%)	35029 (26.6)	55396 (37.4)	18398 (53.5)
Healthy diet, n (%)	75231 (57.1)	79456 (53.7)	16603 (48.3)
Adequate sleep duration, n (%)	103956 (78.9)	109402 (73.9)	22018 (64.1)
Regular physical activity, n (%)	63454 (48.2)	67650 (45.7)	14024 (40.8)
History of diabetes, n (%)	3551 (2.7)	6582 (4.4)	2793 (8.1)
History of cancer, n (%)	10095 (7.7)	13694 (9.3)	3678 (10.7)
History of CVD, n (%)	5726 (4.3)	10176 (6.9)	3961 (11.5)

Continuous variables are expressed as means ± SD, categorical variables are expressed as n (%).

Abbreviations: HbA1c, hemoglobin; n-HDL, non-high-density lipoprotein; CVD, cardiovascular disease.

Smoking was defined as current smoking. Moderate alcohol consumption was defined as ≤14 g/day for women and ≤28 g/day for men, respectively. A healthy diet was defined as participants achieving a diet score ≥4. Adequate sleep duration was defined as participants achieving 7−9 hours of sleep. Regular physical activity was defined as ≥150 min/week of moderate activity, ≥75 min/week of vigorous activity or an equivalent combination.

### 3.2 Association between polysocial risk score and premature mortality

**Fig. 1:**
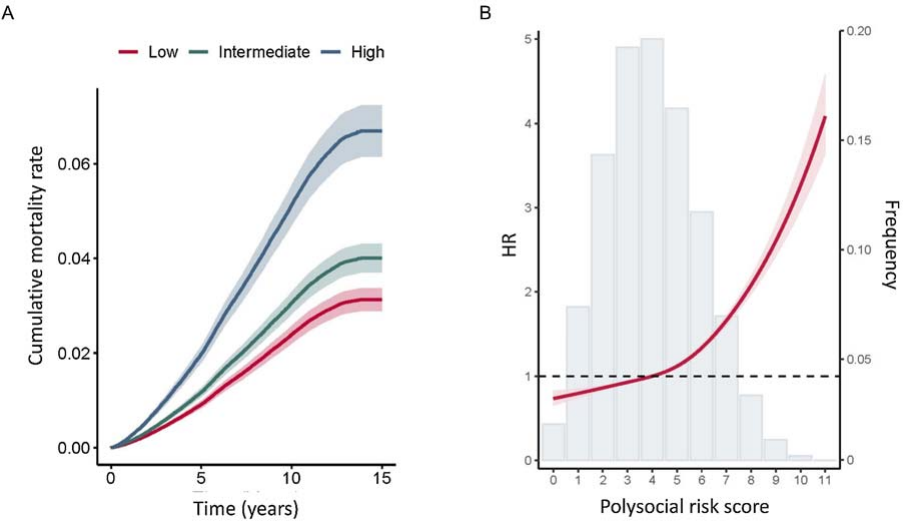
Association of polysocial risk score with incident premature mortality. (A) Cumulative event rate of premature mortality stratified by polysocial risk score category. (B) Distribution of polysocial risk score (frequency, on the right axis) and the associations between polysocial risk score and incident premature mortality (HR, on the left axis). The solid lines are fitted based on Cox-proportional hazard models. The shaded areas show 95% confidential intervals.

The association between polysocial risk score and all-cause premature mortality and cause-specific premature mortality is presented in Table 2. Over a median follow-up of 12.7 years (IQR 12.0–13.3, 3,920,280 person-years), 13,888 cases of premature death were reported. The rates of premature mortality among participants with low, intermediate, and high polysocial risk score were 2.48, 3.66, and 7.24 per 1000 person-years, respectively. After adjustment for covariates, results showed that compared to those with low social risk, the risk of premature death increased by 29% (HR 1.29, 95%CI 1.23–1.34) in the intermediate group, and increased by 118% (HR 2.18, 95%CI 2.06–2.30) in high social risk group (Table 2). Each one increment change in the polysocial risk score was associated with a 15% higher risk of premature death (HR 1.15, 95%CI 1.14–1.16). Kaplan Meier curves showed cumulative mortality rates increased with higher polysocial risk score groups (Fig. 1A). Dose-response relationship analysis showed that polysocial risk score was linearly associated with the risk of premature mortality (Fig. 1B).

**Table 2:** The association of polysocial risk score with all-cause and cause-specific premature mortality.

Cause of death	Polysocial risk score				
	Low (≤3)	Intermediate (4−6)	High (≥7)	Per 1-point	*P* for trend
All-causes					
Model 1 HR (95%CI)	1 (Reference)	1.39 (1.33, 1.44)	2.69 (2.56, 2.81)	1.20 (1.19, 1.21)	<0.001
Model 2 HR (95%CI)	1 (Reference)	1.30 (1.25, 1.36)	2.27 (2.15, 2.40)	1.16 (1.15, 1.17)	<0.001
Model 3 HR (95%CI)	1 (Reference)	1.29 (1.23, 1.34)	2.18 (2.06, 2.30)	1.15 (1.14, 1.16)	<0.001
Cancer					
Model 1 HR (95%CI)	1 (Reference)	1.21 (1.15, 1.28)	1.84 (1.72, 1.96)	1.33 (1.29, 1.38)	<0.001
Model 2 HR (95%CI)	1 (Reference)	1.15 (1.09, 1.21)	1.60 (1.48, 1.72)	1.24 (1.20, 1.29)	<0.001
Model 3 HR (95%CI)	1 (Reference)	1.14 (1.08, 1.20)	1.56 (1.45, 1.69)	1.23 (1.18, 1.27)	<0.001
CVD					
Model 1 HR (95%CI)	1 (Reference)	1.65 (1.50, 1.81)	3.79 (3.41, 4.21)	1.95 (1.84, 2.06)	<0.001
Model 2 HR (95%CI)	1 (Reference)	1.51 (1.37, 1.67)	2.98 (2.64, 3.37)	1.72 (1.62, 1.83)	<0.001
Model 3 HR (95%CI)	1 (Reference)	1.48 (1.34, 1.64)	2.78 (2.46, 3.14)	1.66 (1.56, 1.77)	<0.001
Respiratory diseases					
Model 1 HR (95%CI)	1 (Reference)	2.19 (1.78, 2.68)	7.93 (6.43, 9.79)	2.97 (2.66,3.31)	<0.001
Model 2 HR (95%CI)	1 (Reference)	1.93 (1.55, 2.43)	5.88 (4.63, 7.47)	2.52 (2.23, 2.85)	<0.001
Model 3 HR (95%CI)	1 (Reference)	1.89 (1.51, 2.37)	5.48 (4.31, 6.97)	2.43 (2.15, 2.75)	<0.001
Digestive diseases					
Model 1 HR (95%CI)	1 (Reference)	2.37 (1.87,3.01)	7.63 (5.93, 9.82)	2.84 (2.50, 3.22)	<0.001
Model 2 HR (95%CI)	1 (Reference)	2.49 (1.90, 3.27)	7.19 (5.37, 9.64)	2.72 (2.35, 3.14)	<0.001
Model 3 HR (95%CI)	1 (Reference)	2.44 (1.86, 3.20)	6.71 (5.00, 9.00)	1.62 (2.26, 3.03)	<0.001
Neurodegenerative diseases					
Model 1 HR (95%CI)	1 (Reference)	1.17 (0.93, 1.47)	1.61 (1.19, 2.19)	1.25 (1.08, 1.46)	<0.001
Model 2 HR (95%CI)	1 (Reference)	1.23 (0.96, 1.58)	1.54 (1.07, 2.20)	1.24 (1.04, 1.47)	<0.001
Model 3 HR (95%CI)	1 (Reference)	1.22 (0.95, 1.57)	1.53 (1.07, 2.19)	1.23 (1.04, 1.47)	<0.001

The HRs and 95% CIs were calculated using the Cox proportional hazards model.

Abbreviations: HR = hazard ratio; CI = confidence interval.

Model 1 was adjusted for age and sex.

Model 2 was adjusted for age, sex, ethnicity, BMI, diet, smoking, physical activity, alcohol consumption, and sleep duration.

Model 3 was adjusted for age, sex, ethnicity, BMI, diet, smoking, physical activity, alcohol consumption, sleep duration, HbA1c, n-HDL, and systolic blood pressure.

### 3.3 Subgroup analysis

**Fig. 2:**
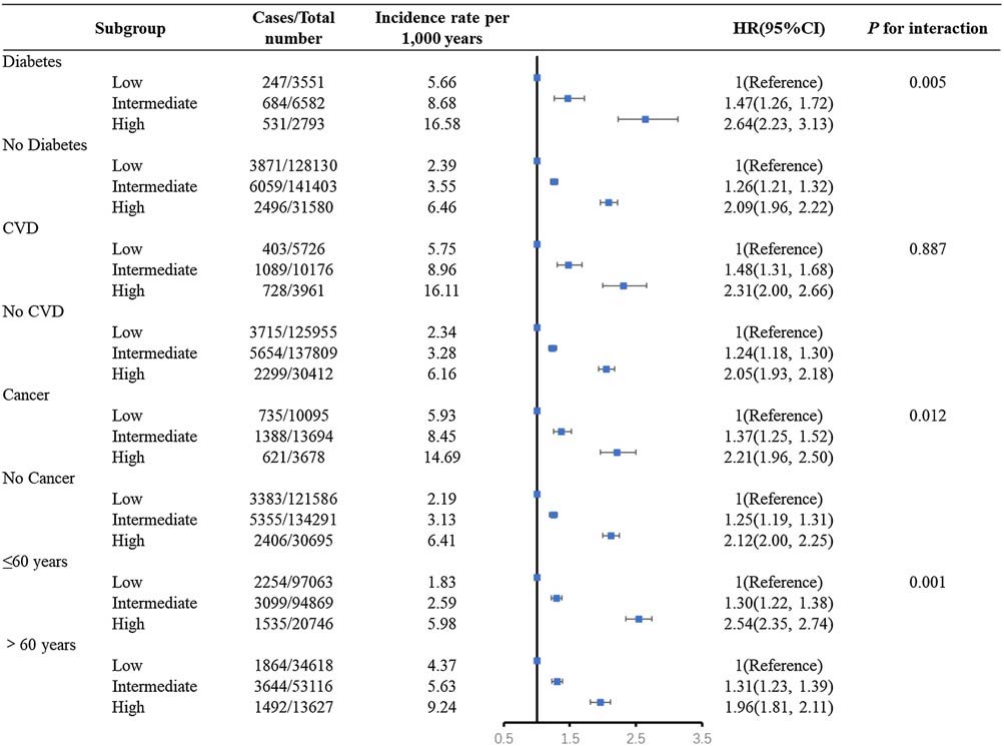
Risk of premature mortality according to polysocial risk score stratified by diabetes, CVD, cancer, and age. The results were adjusted for age, sex, ethnicity, BMI, diet, smoking, physical activity, alcohol consumption, sleep, HbA1c, n-HDL, and systolic blood pressure. HR = hazard ratio; CVD = cardiovascular disease.

Fig. 2 showed that the association between polysocial risk score and premature mortality turned out to be stronger among participants with diabetes (*P* for interaction = 0.005), CVD (*P* for interaction = 0.012), and among those who were younger than 60 years old (*P* for interaction = 0.001). Among participants with diabetes, the hazard ratio for individuals with intermediate and high polysocial risk score were 1.47 (95%CI 1.26, 1.72), and 2.64 (95%CI 2.23, 3.13), respectively. Similarly, the hazard ratio for CVD participants with intermediate and high polysocial risk score were 1.48 (95%CI 1.31, 1.68), and 2.31 (95%CI 2.00, 2.66), respectively. The results were nonsignificant among participants with baseline cancer (*P* for interaction 0.887).

### 3.4 Joint analysis

**Fig. 3:**
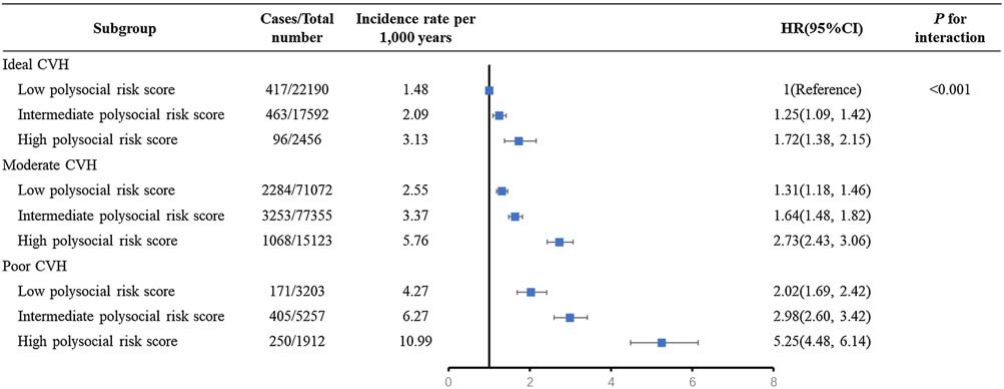
Joint analysis of the associations of CVH status and polysocial risk score with incident premature mortality. Results were adjusted for age, sex, ethnicity, and alcohol consumption. Participants with ideal CVH plus a low polysocial risk score were used as the reference category. CVH = cardiovascular health.

Fig. 3 showed the joint effect of polysocial risk score and CVH on the risk of incident premature death. Participants with poor CVH and a high polysocial risk score had a 5-fold greater risk of premature death than those with ideal CVH and a low polysocial risk score (HR 5.25, 95% CI 4.48–6.14). However, the risk was dramatically attenuated in participants with ideal CVH and a high polysocial risk score (HR 1.67, 95%CI 1.34–2.08). A significant multiplicative interaction between CVH status and polysocial risk score was observed (*P* for interaction = 0.001).

### 3.5 Sensitivity analysis

We performed several sensitivity analyses. First, the associations were not materially changed when using a weighted polysocial risk score (Table S3). Second, when excluding death cases that occurred within the first 3 years of follow-up, similar results persist (Table S4). Third, when further adjusted for CRP and family health history, the association of polysocial risk score and premature mortality remained consistent (Table S4). Fourth, when additionally adjusted for co-morbidities in the regression model, the results were similar to the primary analysis (Table S4). In addition, the associations between individual components of the polysocial risk score and premature mortality were presented in Figure S2.

## 4 Discussion

In this large prospective study among 314,039 UK Biobank participants, we found that polysocial risk score composed of 11 SDOH from different domains were associated with an elevated risk of premature mortality. Participants with a high polysocial risk score had up to 2-fold higher risk of premature mortality compared to those with a low polysocial risk score, independently of major confounders. We further observed that people with poor CVH and high polysocial risk score had the highest risk of premature mortality, and a significant interaction was observed between CVH status and polysocial risk score.

Our results corroborate previous research demonstrating that social determinants play a crucial role in shaping individual’s health outcomes, including premature mortality. However, previous studies mainly focused on one or a few traditional social metrics rather than their combined effects. Emerging social determinants of health, such as social connections and natural environment have rarely been taken into account as a whole. Since social factors matter in aggregate, introducing a polysocial risk score could be an effective approach to assessing the role of multiple social factors in health. The few studies that applied a cumulative risk score approach to evaluate an individual’s social status often set the endpoint to a certain disease or target a specific population group [[26], [27], [28]]. To our knowledge, only one study investigated the impact of cumulative SDOH on all-cause mortality, and the results showed a 2.5-fold higher risk of mortality among individuals in the highest quintile of social disadvantage relative to those in the lowest quintile [29]. However, this study was limited by the relatively short follow-up period and small sample size. Our results were in line with prior studies and added new evidence that a polysocial risk score might be a valuable tool to identify individuals at high risk of premature mortality.

In the stratified analysis, our finding suggested that the association between polysocial risk score and premature death was augmented in individuals with diabetes and CVD, and those younger than 60 years old. These findings underscore the more aggressive strategies of reducing inequities, fostering strong social connections, and supportive environments in younger individuals with non-communicable diseases to reduce mortality risks and promote overall well-being.

Furthermore, we observed a significant interaction between polysocial risk score and CVH that synergistically raised the risk of premature mortality. The increased risk of premature mortality associated with higher polysocial risk score was greater among individuals with a poor CVH than among those with an ideal CVH. Previous studies have largely focused on how health behaviors affected the associations of socioeconomic disparities with mortality and other health outcomes. However, the results remained controversial. In a systematic review, the contribution of health behaviors to socioeconomic inequalities in health varies from 9% to 43% [30]. The largely inconsistent findings may due to different study design, population, and definition of social deprivation and lifestyles. In the present study, we used the “life’s essential 8” algorithm that included an extended measure of healthy behaviors and factors to evaluate an individual’s overall cardiovascular health. Among the study population, less than 20% of participants achieved an ideal CVH. Similar patterns were reported in other research from the US and China, and only about 19.5% and 23.3% of adults were qualified as having ideal CVH in the NHANES and China-PAR cohort, respectively [31,32]. These results, together with ours, highlighted that the percentage of people who have achieved an ideal CVH is far below optimal levels [19]. Besides, it is reported that adults who experienced social adversity were less likely to attain ideal levels of cardiovascular health [33]. Therefore, continued efforts to promote the public’s cardiovascular health is urgent and may help reduce socioeconomic inequalities in premature mortality and promote prolonged life expectancy.

The mechanisms behind the observed association between polysocial risk score and premature mortality are not fully understood. One possible explanation is that social disadvantage may be associated with accelerated biological aging, potentially leading to premature mortality [34]. Another hypothesis is that social stress is associated with increased myelopoiesis, elevated inflammatory and cortisol status, and thus induced mortality [35]. Besides, environmental deprivation has been linked to enhanced oxidative stress, endothelial dysfunction, and compromised immune response, which may affect the population’s life expectancy. Future studies exploring the underlying mechanisms are warranted.

Major strengths of this study include the large sample size, the prospective design with a long-term follow-up, and high-quality data resources. The comprehensive individual-level information in the UK Biobank and the reliable death records through the NHS gave us the opportunity to develop a polysocial risk score. In our knowledge, we were the first to combine 11 social determinants from different domains and measured their aggregated effects on premature mortality. Our results were robust in a series of sensitivity analyses.

There are also several limitations in the current study. First, the mainly self-reported data on social risk factors and lifestyle had the potential of misreporting, under-reporting, and recall bias, however, these data were widely used in prior studies and showed good correlation with objectively measured data. Second, information on the factors used to calculate the polysocial risk score was only documented at baseline, thus, we could not capture the trajectories of these SDOH in the follow-up period. Future studies with repeated data are preferred to validate our findings. Third, despite our efforts to control for various confounding variables, the possibility of unmeasured confounders still remained. We demonstrated the robustness of our findings by performing several sensitivity analyses. Fourth, although UK Biobank provided a wide range of information regarding an individual’s social status, other unmeasured social risk factors such as access to health care and health literacy were not included in the polysocial risk score. Finally, most of the participants in the UK biobank were Caucasians who were prone to be “healthy volunteers”. Thus, it is crucial to interpret our results with caution and generalize it to more diverse populations [36].

## 5 Conclusions

In this population-based cohort study, polysocial risk score was associated with an increased risk of premature mortality, and this risk was exacerbated by poor CVH status. Our findings indicate that reducing social inequities and encouraging people to achieve an ideal CVH are essential to reducing the burden of premature mortality.

## CRediT authorship contribution statement

W.Q.S. and L.L.C conceived the study, cleaned the data, and conducted analyses. W.Q.S wrote the first draft of the manuscript. B.W. and L.L.C. reviewed and edited the manuscript. J.L. and Y.S. contributed to discussion, provided statistical expertise, reviewed and edited the manuscript. N.J.W, and Y.L.L. designed the research, supervised the analyses, reviewed and edited the manuscript. All authors approved the final version of the manuscript.

## Ethics approval and consent to participate

The UK Biobank was approved by the North West Multi-Centre Research Ethics Committee (Ref: 11/NW/0382), and all participants provided written informed consent.

## Funding

This work was supported by 10.13039/501100001809National Natural Science Foundation of China (82120108008), and Shanghai Municipal Health Commission (2022XD017). The funders had no role in the study design, data collection and analysis, publication decision, or manuscript preparation.

## Availability of data and materials

This work was conducted under application number 77740 from the UK Biobank resource. The data that support the findings of this study are publicly available on application to the UK Biobank (www.ukbiobank.ac.uk/).

## Declaration of competing interest

The authors declare that they have no competing interests.
