# Supplementary material for: Association of polysocial risk score, cardiovascular health status, and the risk of premature mortality: Findings from the UK Biobank
Source: J Nutr Health Aging. 2025 Mar 8;29(5):100527. doi: 10.1016/j.jnha.2025.100527 (PMC12180011; doi:10.1016/j.jnha.2025.100527)
Supplement: Supplementary file 1 [file mmc1.docx]

**Supplementary material**

**Association of polysocial risk score, cardiovascular health, and the risk of premature mortality: findings from the UK Biobank**

**Table S1.** Description of social determinants of health used in the present study.

**Table S2.** Calculation of SDOHs included in the polysocial risk score.

**Table S3.** Associations of a weighted polysocial risk score with premature mortality.

**Table S4.** Sensitivity analysis of the association between polysocial risk score and incidence of premature mortality.

**Figure S1.** The flow chart of participants' inclusion and exclusion.

**Figure S2.** Associations of individual social risk factors with premature mortality.

**Table S1. Description of social determinants of health used in the present study.**

| **Social determinant of health** | **Field ID** | **Description** | **Definition**  **(1= at risk, 0=reference)** |
| --- | --- | --- | --- |
| **Socioeconomic status** |  |  |  |
| Low household income | 738 | ACE touchscreen question "What is the average total income before tax received by your HOUSEHOLD?" | 1=less than 31,000  0=more than 31,000 |
| Low education attainment | 6138 | ACE touchscreen question "Which of the following qualifications do you have? | 1=college  0=lower than college |
| Poor education quality | 26414 26431 26421 | This domain measures the extent of deprivation in terms of education, skills and training in an area. | 1=above median  0=below median |
| Not in paid employment | 6142 | ACE touchscreen question "Which of the following describes your current situation?" | 1=others  0=in paid employment or self-employed |
| **Psychosocial factors** |  |  |  |
| Living alone | 709 | ACE touchscreen question "Including yourself, how many people are living together in your household? (Include those who usually live in the house such as students living away from home during term, partners in the armed forces or professions such as pilots)" | 1=living alone  0=more than one |
| Lack of social support | 2110 | ACE touchscreen question "How often are you able to confide in someone close to you?" | 1=less than once a week  0= more than once a week |
| Social inactivity | 6160 | ACE touchscreen question "Which of the following do you attend once a week or more often? | 1= none  0= any |
| Social isolation | 1031 | ACE touchscreen question "How often do you visit friends or family or have them visit you?" | 1=once a week or less  0=more than once a week |
| Emotional distress | 6145 | ACE touchscreen question "In the last 2 years have you experienced any of the following (Illness, injury, bereavement, stress in last 2 years) ? | 1=any  0=none |
| Psychiatrist visit | 2100 | ACE touchscreen question "Have you ever seen a psychiatrist for nerves, anxiety, tension or depression?" | 1=yes  0=no |
| **Neighborhood and living environment** |  |  |  |
| Area-level material deprivation | 22189 | A measure of material deprivation | 1=above median  0=below median |
| High local crime rate | 26416  26434 26425 | This domain measures the rate of recorded crime in an area for four major crime types (Violence, Burglary, Theft, Criminal damage) representing the risk of personal and material victimization at a small area level. | 1=above median  0=below median |
| Natural environnement | 24506 | The percentage of the home location buffer classed as 'Natural Environment' in the Land Cover Map (LCM) 2007, and with home location data buffered at 1000m. | 1=below median  0=above median |
| Air pollution | 24006 | PM10 (particulate matter with diameter less than or equal to 2.5 micrometers) | 1=above median  0=below median |
| Noise pollution | 24024 | Average 24-hour sound level of noise pollution | 1=above median  0=below median |

**Table S2. Calculation of SDOHs included in the polysocial risk score.**

| **Social determinant of health** | **HR (95%CI)** | **p** |
| --- | --- | --- |
| *Low education | 1.06(1.02, 1.10) | 0.007 |
| *Low education quality | 1.11(1.07, 1.16) | <0.001 |
| *Low household income | 1.17(1.12, 1.23) | <0.001 |
| *Townsend | 1.06(1.01, 1.10) | 0.012 |
| *Not employed | 1.31(1.25, 1.37) | <0.001 |
| *Living alone | 1.26(1.21, 1.32) | <0.001 |
| *Social inactivity | 1.09(1.05, 1.14) | <0.001 |
| *Social isolation | 1.14(1.09, 1.19) | <0.001 |
| Lack of social support | 1.01(0.97, 1.05 | 0.711 |
| *Emotional distress | 1.33(1.28, 1.38) | <0.001 |
| *Psychiatrist visit | 1.20(1.14, 1.27) | <0.001 |
| High local crime rate | 1.02(0.97, 1.06) | 0.453 |
| *P.M. 2.5 pollution | 1.08(1.03, 1.13) | 0.001 |
| Natural environment | 0.98(0.94, 1.03) | 0.453 |
| Exposure to noise pollution | 0.99(0.96, 1.03) | 0.787 |

The HRs and 95% CIs were calculated using the Cox hazard model. The model was adjusted for all covariables including age, sex, ethnicity, BMI, diet, smoking, physical activity, alcohol consumption, sleep duration, HbA1c, n-HDL, and systolic blood pressure.

Social determinants of health with a * marker were included in the calculation of the polysocial risk score.

**Table S3. Associations of a weighted polysocial risk score with incidence premature mortality.**

|  | **Weighted polysocial risk score** | | | |  |
| --- | --- | --- | --- | --- | --- |
|  | T1 | T2 | T3 | per 1-SD increment | *P* for trend |
| Cases | 2437 | 3971 | 7480 |  |  |
| Person-years | 1,132,552 | 1,374,360 | 1,413,368 |  |  |
| HR (95%CI) ^a^ | 1(Reference) | 1.22(1.16, 1.28) | 2.03(1.94, 2.13) | 1.45(1.42, 1.47) |  |
| HR (95%CI) ^b^ | 1(Reference) | 1.17(1.11, 1.24) | 1.81(1.72, 1.91) | 1.37(1.34, 1.39) | <0.001 |
| HR (95%CI) ^c^ | 1(Reference) | 1.16(1.10, 1.23) | 1.76(1.68, 1.86) | 1.35(1.32, 1.37) | <0.001 |

T1= first tertile, T1= second tertile, T3= third tertile, SD= standard deviation, HR = hazard ratio

^a^Adjusted for age, and sex.

^b^Adjusted for age, sex, ethnicity, BMI, diet, smoking, physical activity, alcohol consumption, and sleep duration.

^c^Adjusted for age, sex, ethnicity, BMI, diet, smoking, physical activity, alcohol consumption, sleep duration, HbA1c, n-HDL, and systolic blood pressure.

**Table S4. Sensitivity analysis of the association of polysocial risk score with incidence of premature mortality.**

|  | **Polysocial risk score** | | |  |
| --- | --- | --- | --- | --- |
|  | Low | Intermediate | High | *P* for trend |
| ***Excluding cases that occurred in the first 3 years*** |  |  |  |  |
| Cases | 3576 | 5649 | 2491 |  |
| Person-years | 1,658,263 | 1,840,915 | 417,322 |  |
| Adjusted HR (95%CI)^a^ | 1(Reference) | 1.25(1.19, 1.30) | 2.10(1.98, 2.23) | <0.001 |
| ***Additionally adjusted for CRP, family history of diseases*** |  |  |  |  |
| Cases | 4118 | 6743 | 3027 |  |
| Person-years | 1,659,247 | 1,842,805 | 418,228 |  |
| Adjusted HR (95%CI)^b^ | 1(Reference) | 1.25(1.19, 1.31) | 2.07(1.96, 2.20) | <0.001 |
| ***Additionally adjusted for co-morbidities*** |  |  |  |  |
| Cases | 4118 | 6743 | 3027 |  |
| Person-years | 1,659,247 | 1,842,805 | 418,228 |  |
| Adjusted HR (95%CI)^c^ | 1(Reference) | 1.26 (1.20, 1.31) | 2.05 (1.94, 2.17) | <0.001 |

^a^Adjusted for age, sex, ethnicity, BMI, diet, smoking, physical activity, alcohol consumption, sleep duration, HbA1c, n-HDL, and systolic blood pressure.

^b^Adjusted for age, sex, ethnicity, BMI, diet, smoking, physical activity, alcohol consumption, sleep duration, HbA1c, n-HDL, systolic blood pressure, CRP, and family history of diseases. Information on family history of diseases was obtained from Data fields 20107, and 20110.

^c^Adjusted for age, sex, ethnicity, BMI, diet, smoking, physical activity, alcohol consumption, sleep duration, HbA1c, n-HDL, systolic blood pressure, and co-morbidities (diabetes, cardiovascular diseases, and cancer).


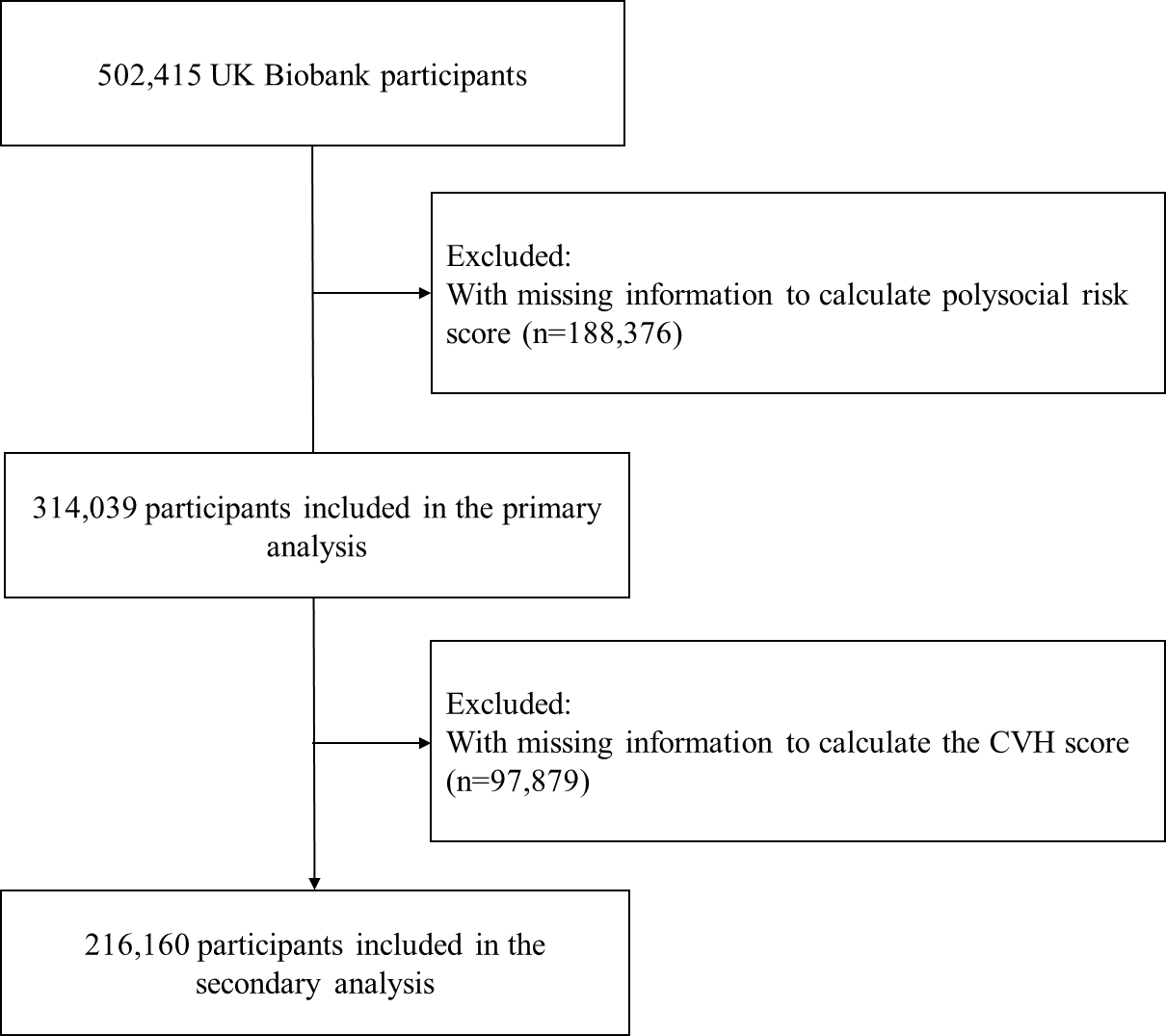


**Figure S1. The flow chart of participants' inclusion and exclusion.**

**
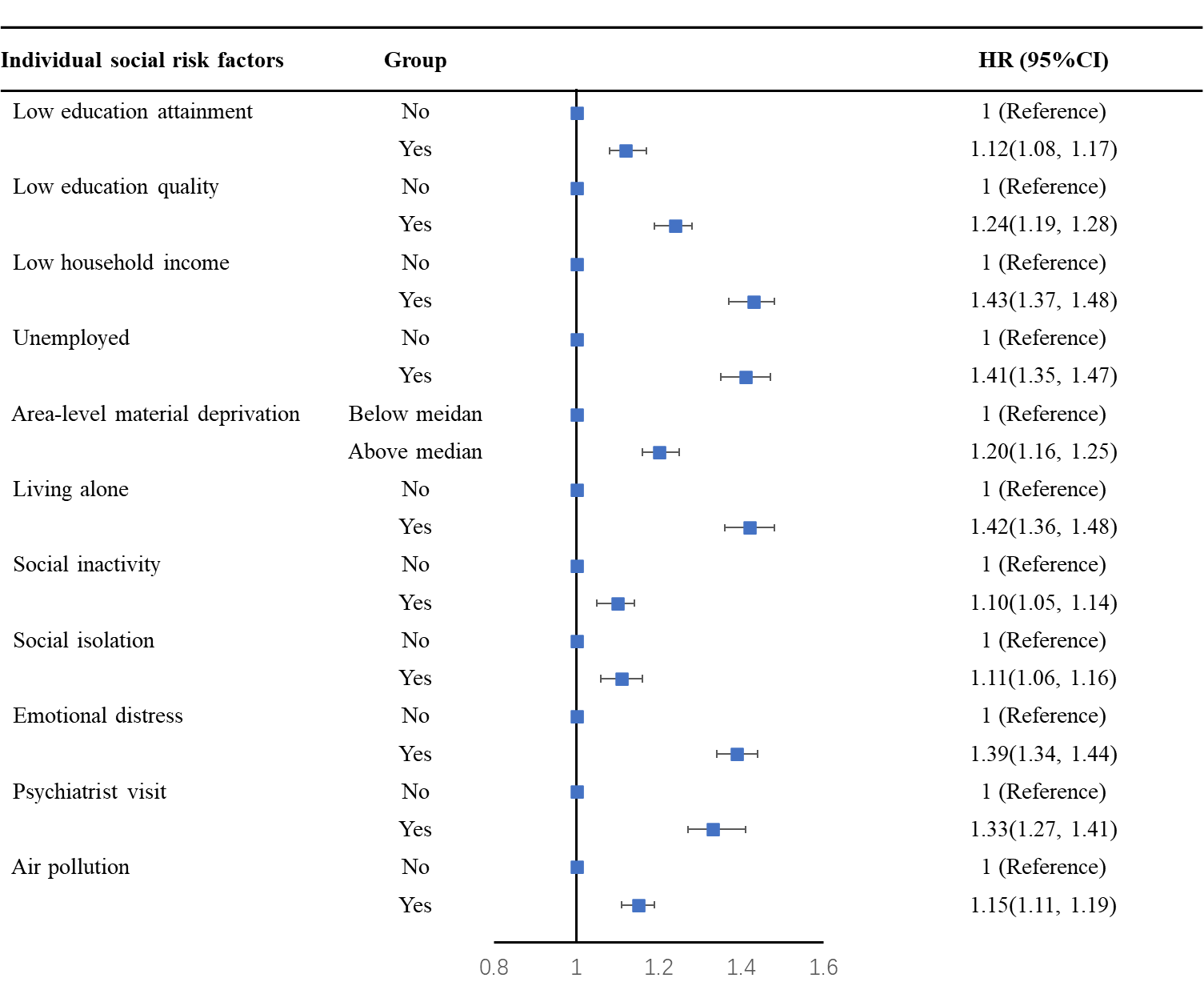
**

**Figure S2. Associations of individual social risk factors with premature mortality.**
